# Decreased efficacy of drugs targeting the vascular endothelial growth factor pathway by the epigenetic silencing of *FLT1* in renal cancer cells

**DOI:** 10.1186/s13148-015-0134-9

**Published:** 2015-09-16

**Authors:** Jee Yeon Kim, Junha Hwang, Seo Hyun Lee, Hyo Jin Lee, Jaroslav Jelinek, Hyeseon Jeong, Jae Sung Lim, Jin Man Kim, Kyu Sang Song, Byung Hoon Kim, Sukhoon Lee, Jei Kim

**Affiliations:** Neuroepigenetics Laboratory and Department of Neurology, Hospital and School of Medicine, Chungnam National University, Daejeon, South Korea; Department of Information and Statistics, College of Natural Science, Chungnam National University, Daejeon, South Korea; Department of Internal Medicine, Cancer Research Institute, Hospital and School of Medicine, Chungnam National University, Daejeon, South Korea; Fels Institute for Cancer Research and Molecular Biology, Temple University, Philadelphia, PA USA; Department of Urology, Hospital and School of Medicine, Chungnam National University, Daejeon, South Korea; Department of Pathology, Hospital and School of Medicine, Chungnam National University, Daejeon, South Korea; Department of Urology, Hospital and School of Medicine, Keimyung University, Daegu, South Korea; Department of Neurology, Chungnam National University Hospital, 282 Moonhwa-ro, Joong-gu Daejeon, 301–721, South Korea

**Keywords:** Vascular endothelial growth factor, Vascular endothelial growth factor receptor, Anti-vascular endothelial growth factor antibody, Tyrosine kinase inhibitor, Sunitinib, Axitinib, Renal cancer

## Abstract

**Background:**

The vascular endothelial growth factor (VEGF)-VEGF receptor (VEGFR) signaling pathway is involved in cancer-related biological functions and is a therapeutic target in cancer. However, the influence of epigenetic regulation of VEGF-VEGFR signaling-related genes remains unclear. Here, we evaluated the effects of *FLT1* and *KDR* promoter hypermethylation combined with drugs targeting VEGF-VEGFR signaling on cancer-related phenotypes in renal cancer cells (RCCs) and examined changes in *FLT1* and *KDR* promoter hypermethylation in tissues from patients with renal cancer.

**Results:**

In vitro experiments were performed to evaluate the effects of beavacizumab (an anti-VEGF antibody), an anti-FLT1 peptide, an anti-KDR antibody, and the VEGFR tyrosine kinase inhibitors (TKIs) sunitinib and axitinib in 13 RCC lines with different levels of *FLT1* and/or *KDR* promoter methylation and in 2 FLT1 or KDR in vitro knockdown models. The synergistic effects of sunitinib and axitinib treatment were also evaluated in four RCC lines having different levels of *FLT1* and/or *KDR* methylation. In our in vitro experiments, bevacizumab and an anti-KDR antibody did not affect the proliferation of RCCs having *FLT1* and/or *KDR* hypermethylation. In contrast, in RCCs with *FLT1* hypermethylation, proliferation inhibition was counteracted by treatment with an anti-FLT1 peptide and both VEGF-TKIs (sunitinib and axitinib). Demethylation with sunitinib or axitinib synergistically increased proliferation inhibition in the RCCs exhibiting *FLT1* hypermethylation. Using in vitro *FLT1* or *KDR* knockdown models, decreased proliferation inhibition following anti-FLT1 peptide, sunitinib, and axitinib treatment was observed only in *FLT1*-knockdown cells. In patients with renal cancer who received sunitinib, *FLT1* promoter methylation was higher in renal cancer tissues from eight nonresponders (stable or progressive disease assessed by the Response Evaluation Criteria in Solid Tumors) than in cancer tissues from five responders (complete response or partial response).

**Conclusions:**

The present data showed that hypermethylated *FLT1* was important for the efficacy of anti-VEGF/VEGFR drugs targeting FLT1 or intracellular VEGFR signaling. *FLT1* hypermethylation causing alterations of FLT1 function could serve as a useful biomarker for predicting changes in *FLT1* status in RCCs.

**Electronic supplementary material:**

The online version of this article (doi:10.1186/s13148-015-0134-9) contains supplementary material, which is available to authorized users.

## Background

Vascular endothelial growth factor (VEGF)-VEGF receptor (VEGFR) signaling is a critical step for autocrine mitogenesis and paracrine angiogenesis during tumor growth [1]. A variety of drugs targeting VEGF-VEGFR signaling (anti-VEGF/VEGFR drugs) have been successful in halting or regressing tumor growth in various in vitro and in vivo studies [2]. However, these anti-VEGF/VEGFR drugs have only modest effects in patients with cancer [2]. Although studies have evaluated the anti-angiogenic mechanisms of anti-VEGF/VEGFR drugs in order to understand the lack of success in the clinical setting [3], additional studies are needed to further elucidate other mechanisms supporting these observations.

For anti-VEGF/VEGFR drugs to be effective in cancer cells, active VEGF-VEGFR signaling should occur in cancer cells, as occurs in endothelial cells. In a previous study, endothelial cells showed no epigenetic gene silencing of *VEGF*, *VEGFR1* (*FLT1*), or *VEGFR2* (*KDR*), and cell proliferation could be inhibited by treatment with an anti-VEGF antibody, an anti-KDR antibody, or VEGF tyrosine kinase inhibitors (TKIs) [4]. However, in some cancer cells, changes in intracellular VEGF-VEGFR signaling occur due to epigenetic gene silencing of *FLT1* and *KDR* [5]. Cell lines having epigenetic gene silencing of both *FLT1* and *KDR* show insufficient inhibition of proliferation after treatment with VEGF-TKIs [4]. While a previous study showed evidence that intact VEGF-VEGFR signaling is necessary for the successful effects of anti-VEGF/VEGFR drugs, [4] the study was conducted using cancer cells that originated from various human tissues, and the individual roles of *FLT1* or *KDR* epigenetic gene silencing were not appropriately evaluated. Therefore, the potential success or failure of anti-VEGF/VEGFR drugs in cancer cells originating from different tissue types and with different levels of *FLT1* or *KDR* methylation remains unclear.

In the present study, we aimed to analyze whether epigenetic alterations in *FLT1* and/or *KDR* are related to the anti-cancer effects of drugs targeting VEGF-VEGFR signaling in renal cancer cells (RCCs) and in tissues collected from renal cancer patients.

## Results

### Methylation of the *FLT1* and *KDR* promoters in RCC lines

**Fig. 1:**
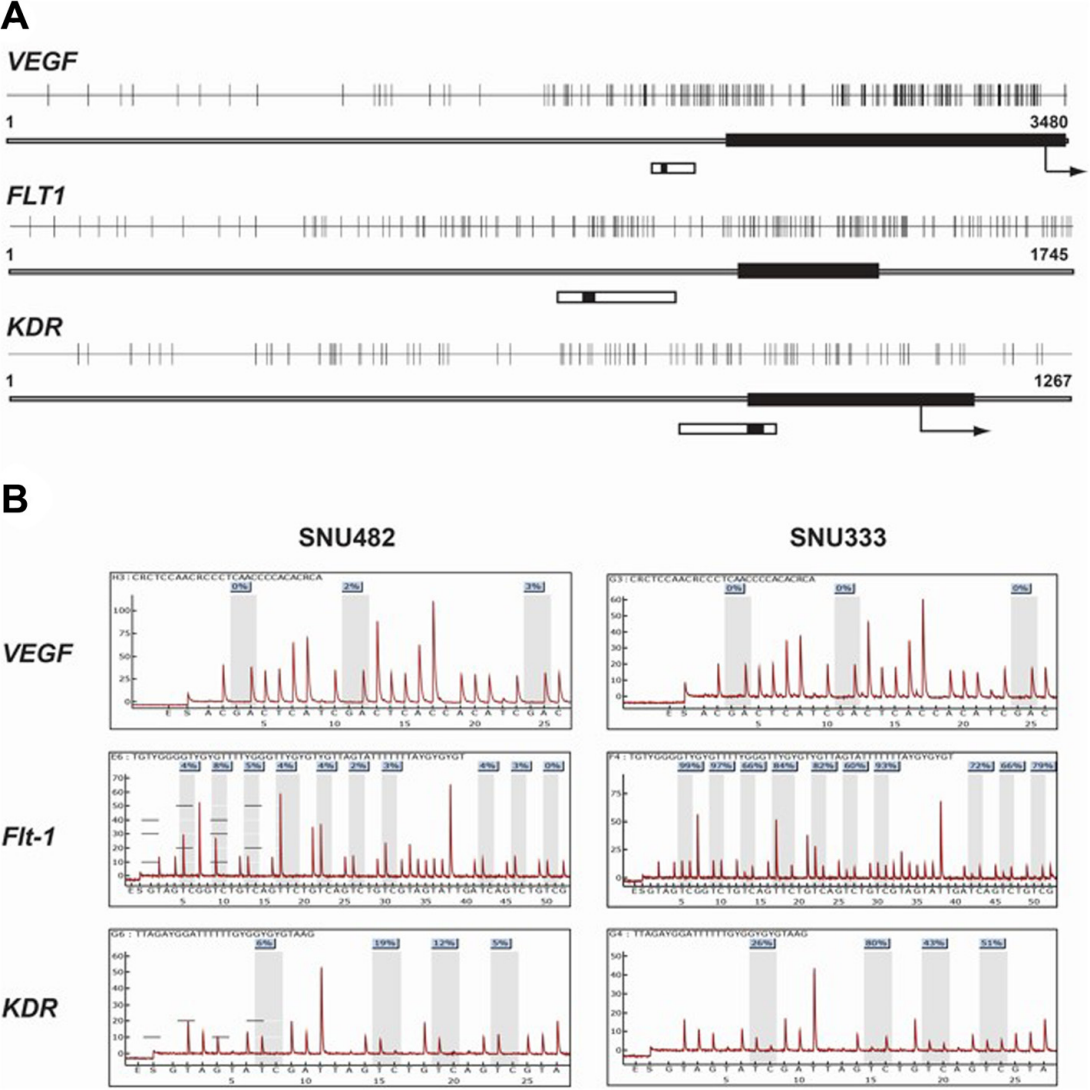
Promoter CpG islands (**a**) and *VEGF*, *FLT1*, and *KDR* pyrosequencing in 2 RCC lines (**b**). *Closed bars*, exon 1 region for each gene; *arrows*, translation start site; *open bars*, the region targeted for pyrosequencing; *black segments within open bars*, locations of sequencing primers for each gene

**Fig. 2:**
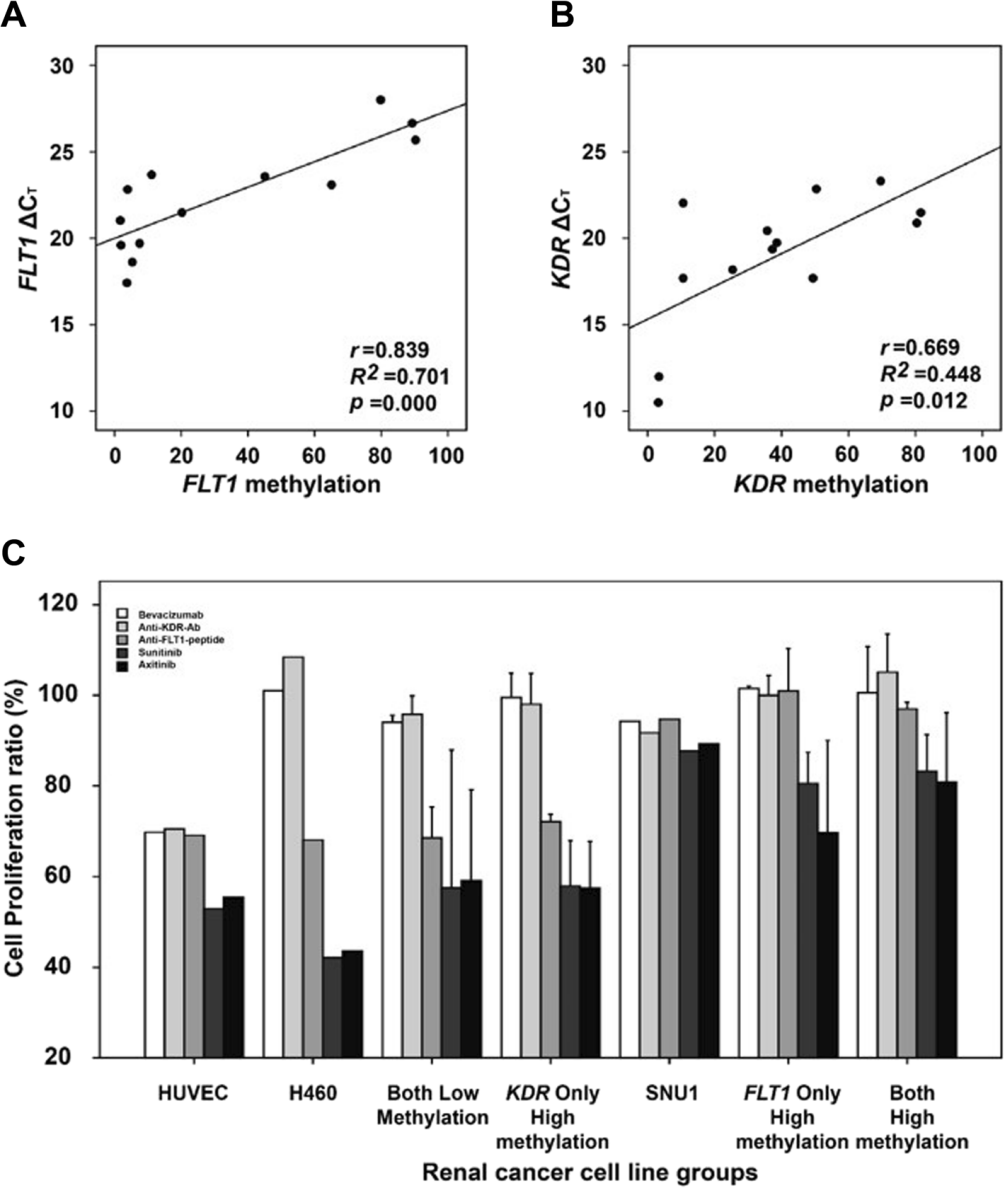
Expression changes and anti-VEGF/VEGFR drug efficacies associated with *FLT1* and *KDR* methylation changes. Analysis of gene expression of *FLT1* (**a**) and *KDR* (**b**) in 13 RCC lines. Evaluation of the effects of bevacizumab, an anti-FLT1 peptide, an anti-KDR antibody, sunitinib, and axitinib on RCC line proliferation was classified according to the hypermethylation status of *FLT1* and/or *KDR* (**c**). H460 cells and SNU1 cells were used as control cell lines that lacked or high methylation of either gene, respectively . The *error bars* show standard errors

First, we examined the levels of *VEGF*, *FLT1*, and *KDR* promoter methylation in select cell lines by pyrosequencing to target a sequence in promoter region of each gene (Fig. 1a, b). Human umbilical vein endothelial cells (HUVECs) showed less than 4 % methylation of *VEGF*, *FLT1*, and *KDR* (Table 1). In contrast, 13 RCC lines that were tested showed less than 1 % promoter methylation of *VEGF* but variable methylation (from 2 to 90 %) for *FLT1* or *KDR* (Table 1) . The increase in promoter methylation for *FLT1* (*r* = 0.839, *R*^*2*^ = 0.701, *p* = 0.000) and *KDR* (*r* = 0.669, *R*^*2*^ = 0.448, *p* = 0.012) corresponded to an increase in ΔC_T_ values in real-time reverse transcription polymerase chain reaction (RT-PCR) experiments (Fig. 2a, b).

### Methylation of the *FLT1* and *KDR* promoters in renal cancer tissues and in sequences deposited in The Cancer Genome Atlas (TCGA) database

**Fig. 3:**
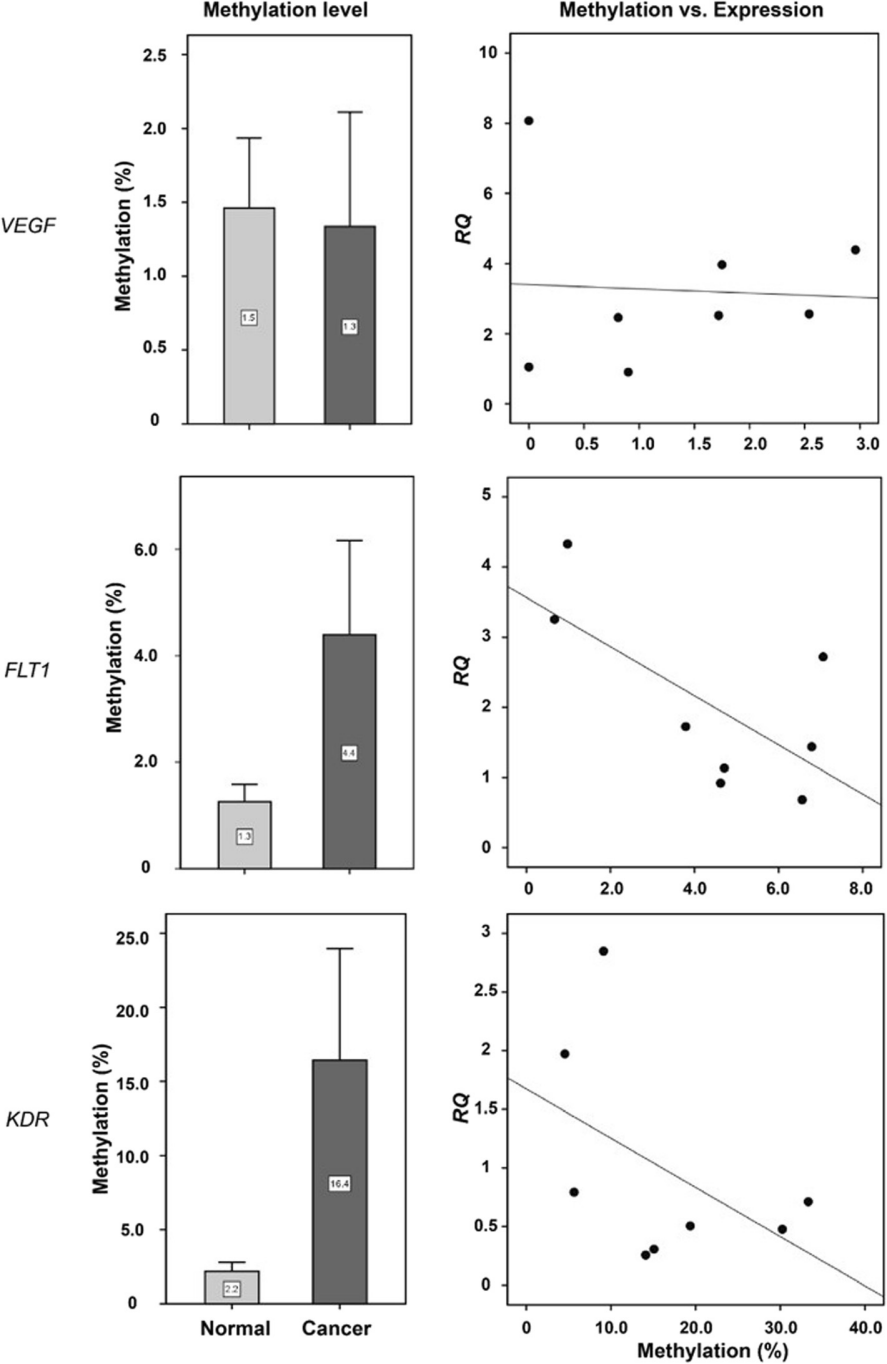
*VEGF*, *FLT1*, and *KDR* promoter methylation and expression differences between normal and cancer tissues. *Normal*, normal tissues; *cancer*, cancer tissues collected from eight renal cancer patients

To evaluate whether epigenetic gene silencing occurs in renal cancer tissue, we analyzed the relationship between promoter methylation and expression of *VEGF*, *FLT1*, and *KDR* in normal vs. cancer tissues collected from eight renal cancer patients (Fig. 3). Normal and cancer tissues showed less than 2 % promoter methylation for *VEGF* (*p* = 0.641). However, *FLT1* (normal, 1.3 %; cancer tissue, 4.4 %; *p* = 0.023) and *KDR* (2.2 % vs. 16.4 %; *p* = 0.008) methylations were significantly higher in cancer tissues, compared to normal tissues (Fig. 3). Next, we tested for associations between expression and methylation of *FLT1* and *KDR* in renal cancer tissues. This was done by performing correlation analysis between the reciprocal of the percent methylation of either promoter and the relative quantity (RQ) of gene expression to determine statically significant linear correlation coefficients. The corresponding regression equations were as follows:

$$ \mathrm{R}\mathrm{Q}\kern0.5em \mathrm{of}\kern0.5em FLT1=1.3302+1.7336\kern0.5em *\frac{1}{FLT1\kern0.5em \mathrm{methylation}}\left(p=0.0669\right) $$

$$ \mathrm{R}\mathrm{Q}\ \mathrm{of}\kern0.5em KDR = 7.9906+0.3335\kern0.5em *\frac{1}{KDR\kern0.5em \mathrm{methylation}}\left(p = 0.048\right) $$

**Fig. 4:**
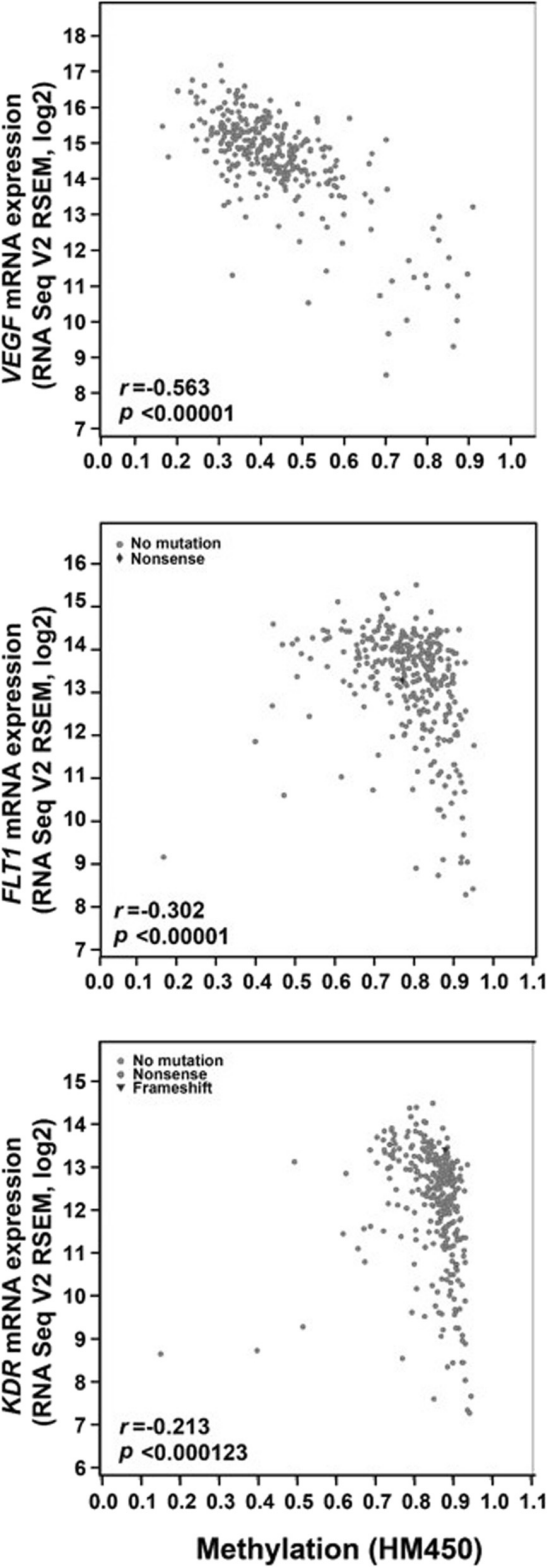
Expression differences associated with promoter methylation changes of the *VEGF*, *FLT1*, and *KDR* genes in TCGA. The query process was performed using the cBioPortal online tools (www.cbioportal.org)

Analysis of data in TCGA deposited under the category “renal clear cell carcinoma” revealed that the expression of *VEGFA* (Spearman correlation *r* = −0.563, *p* < 0.00001), *FLT1* (*r* = −0.302, *p* < 0.00001), and *KDR* (*r* = −0.213, *p* = 0.000123) inversely correlated with methylation of their respective promoters, based on data from 320 clear cell RCC samples in TCGA (Fig. 4).

### The effects of anti-VEGF/VEGFR drugs varied according to the promoter methylation status of *FLT1* or *KDR*

The proliferation of HUVECs was effectively inhibited after treatment with five different anti-VEGF/VEGFR drugs targeting different aspects of the VEGF-VEGFR signaling pathway (an anti-VEGF antibody, bevacizumab, an anti-KDR-antibody, an anti-FLT1 peptide, and 2 VEGF-specific TKIs, sunitinib and axitinib) for 72 h (Fig. 2c). H460 cells, a control cell line having low methylation of the *FLT1* and *KDR* promoters, showed no proliferation inhibition with bevacizumab or treatment with an anti-KDR antibody. However, increased proliferation inhibition was observed by treatment with the anti-FLT1 peptide, sunitinib, or axitinib (Fig. 2c). SNU1 cells, a control cell line exhibiting high methylation of the *FLT1* and *KDR* promoters, showed no proliferation inhibition following treatment with any of these agents (Fig. 2c).

The 13 RCC lines tested were classified into four groups based on the methylation level of *FLT1* and/or *KDR*: low methylation of both genes (both low methylation; SNU482 and SNU228 cells), high methylation of *FLT1* and low methylation of KDR (high *FLT1*/low *KDR* methylation; SN12C and SN12PM6 cells), low methylation of *FLT1* and high methylation of *KDR* (low *FLT1*/high *KDR* methylation; A704, ACHN, Caki-1, and SNU1272 cells), and high methylation of both genes (both high methylation; SNU333, SNU349, A498, and SNU267 cells), as detailed in Table 1. The cutoff methylation levels required for classification as low or high methylation was 15 % of pyrosequencing results for *FLT1* or *KDR*. RCCs showed different proliferation inhibition patterns according to the levels of *FLT1* and *KDR* methylation observed following treatment with the 4 VEGF different pathway inhibitors (Fig. 2c). Cells exhibiting no methylation of *FLT1* and *KDR* or low *FLT1*/high *KDR* methylation exhibited proliferation inhibition patterns similar to that of H460 cells, i.e., no significant proliferation inhibition caused by treatment with bevacizumab and the anti-KDR antibody, but proliferation inhibition was caused by the anti-FLT1 peptide and VEGF-TKIs (sunitinib and axitinib). In contrast, cells exhibiting high methylation of both genes or high *FLT1*/low *KDR* methylation had proliferation inhibition patterns similar to that of SNU1 cells, i.e., no significant proliferation inhibition by any of the tested anti-VEGF/VEGFR drugs.

**Table 1:** Groups of renal cancer cell lines by the promoter methylation status of *FLT1* and *KDR*

Tissue origin	Cell line	Methylation (%)			Group
		VEGF	FLT1	KDR	
EC	HUVEC	2	1	4	Both low (EC control)
Stomach	SNU1	0	93	83	Both high (cancer cell control)
Lung	H460	0	3	8	Both low (cancer cell control)
Kidney	SNU482	0	4	11	Both low
	SNU228	0	2	11	Both low
	SN12C	0	65	3	High Flt1/low KDR
	SN12PM6	0	45	3	High Flt1/low KDR
	A704	0	11	82	Low Flt1/high KDR
	ACHN	0	2	70	Low Flt1/high KDR
	Caki-1	0	5	49	Low Flt1/high KDR
	SNU1272	0	4	39	Low Flt1/high KDR
	Caki-2	0	8	25	Low Flt1/high KDR
	SNU333	1	80	50	Both high
	SNU349	0	89	80	Both high
	A498	0	90	36	Both high
	SNU267	1	20	37	Both high

*EC* endothelial cell, *HUVEC* human umbilical vein endothelial cell, *both low* low methylation (<15 %) of both *FLT1* and *KDR, high Flt1/low KDR* high methylation (>15 %) of *FLT1* and low methylation of *KDR*, *low FLT1/high KDR* low methylation of *FLT1* and high methylation of *KDR, both high* high methylation of both *FLT1* and *KDR*

Generalized linear mixed model (GLMM) analysis was performed to evaluate whether high methylation *FLT1* or *KDR* was related to proliferation inhibition by any of the five drugs in RCCs. This analysis showed that proliferation inhibition of each drug tested was not significantly different between RCC cells exhibiting low and high *KDR* methylation (bevacizumab: *t* = 0.44, *p* = 0.6612; anti-KDR antibody: *t* = 0.67, *p* = 0.5096; anti-FLT1 peptide: *t* = 0.00, *p* = 0.9987; sunitinib: *t* = 0.28, *p* = 0.7790; axitinib: *t* = 0.84, *p* = 0.4077). However, in RCCs with high *FLT1* methylation, the proliferation-inhibitory effects of the anti-FLT1 peptide (*t* = 5.28, *p* < 0.0001), sunitinib (*t* = 4.77, *p* < 0.0001), and axitinib (*t* = 3.78, *p* = 0.0005) were significantly decreased compared to RCCs with low *FLT1* methylation, while growth inhibition caused by bevacizumab (*t* = 0.55, *p* = 0.5869) and the anti-KDR antibody (*t* = 1.16, *p* = 0.2519) was similar between cells exhibiting low and high *FLT1* methylation.

### Different synergistic proliferation-inhibitory effects of sunitinib and axitinib after demethylation, according to the promoter methylation status of *FLT1* and/or *KDR*

**Fig. 5:**
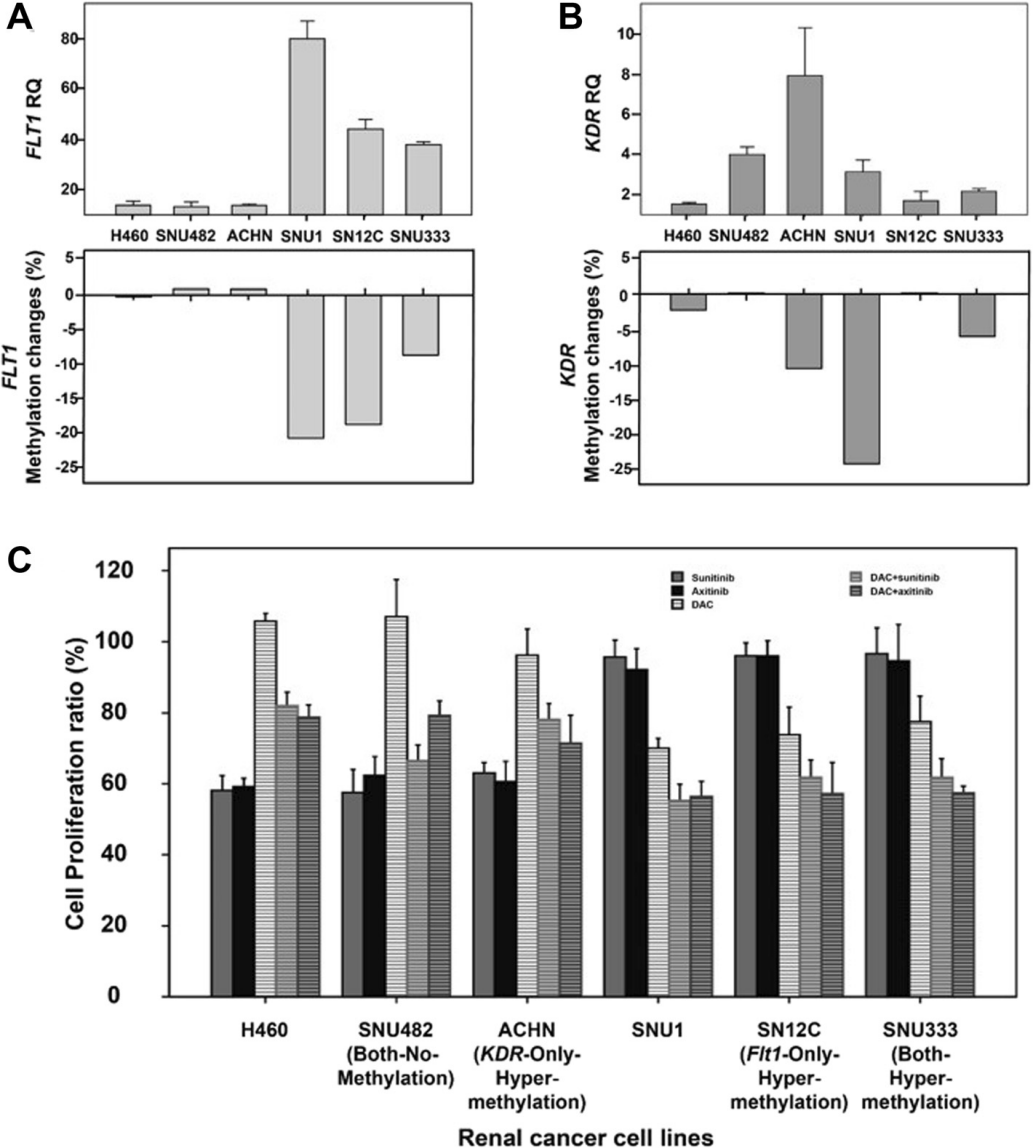
*FLT1* and *KDR* expression and the proliferation-inhibitory effects of sunitinib or axitinib after demethylation treatment. Promoter methylation and gene expression were evaluated after demethylation treatment in RCC lines. Changes in expression (RQ) and methylation (%) in *FLT1* (**a**) and *KDR* (**b**) after demethylation using DAC, a demethylating agent. Proliferation of RCC lines following treatment with sunitinib or axitinib and/or DAC(**c**). The *error bars* show standard errors

Four RCC lines (SNU482, having low methylation of both *FLT1* and *KDR*; SN12C, having high *FLT1*/low *KDR* methylation; ACHN, having low *FLT1*/high KDR methylation; and SNU333, having high methylation of both *FLT1* and *KDR*) were used for demethylation experiments. All four cell lines showed no significant changes in demethylation (0–2 %) or expression of *VEGF* (RQ, 0.3–0.8) after 5-aza-2′-deoxycytidine (DAC) treatment (data not shown)). However, after DAC treatment, SNU333 cells showed significant demethylation and increased *FLT1* expression (methylation change, −9 %; RQ, 28.0) and *KDR* expression (methylation change, −6 %; RQ, 2.9); ACHN cells showed significant changes in methylation and expression of *KDR* (methylation change, −11 %; RQ, 17.3), and SN12C cells showed significant changes in methylation and expression of *FLT1* (methylation change, −19 %; RQ, 34.1; Fig. 5a, b). SNU482 cells showed marked increases in *KDR* expression (RQ, 88.1); however, no significant demethylation was observed for either *FLT1* (methylation change, 1 %) or *KDR* (0.1 %; Fig. 5a, b).

The control cell line H460, which has low methylation of *FLT1* and *KDR*, showed a marked inhibition of proliferation following treatment with sunitinib or axitinib alone. However, combination treatment with DAC and either sunitinib or axitinib showed a lower synergistic effect on proliferation inhibition in H460 cells than in SNU1 cells (Fig. 5c). In contrast, SNU1 cells having high *FLT1* and *KDR* promoter methylation, showed no proliferation inhibition following treatment with sunitinib or axitinib. However, the combination treatment of DAC with sunitinib or axitinib exerted marked synergistic effects on proliferation inhibition. In RCCs, the proliferation inhibition of both SNU482 (low methylation of *FLT1* and *KDR*) and ACHN (low *FLT1*/high *KDR* methylation) cells was promoted by sunitinib or axitinib treatment, which was enhanced to a lesser degree by demethylation, similar to H460 cells. Conversely, the proliferation of SN12C (high *FLT1*/low *KDR* methylation) and SNU333 (high methylation of *FLT1* and *KDR*) cells was synergistically inhibited by combination treatment with DAC and either sunitinib or axitinib, similar to SNU1 cells, although sunitinib or axitinib treatment alone did not significantly inhibit proliferation (Fig. 5c).

Three-factor analysis was used to evaluate the relationship between *FLT1* or *KDR* hypermethylation with the effects of combination treatment of demethylation with VEGF-TKIs in RCCs. This analysis revealed that RCCs exhibiting low *FLT1* methylation were more sensitive to sunitinib (*t* = 11.68, *p* < 0.0001) or axitinib (*t* = 10.96, *p* < 0.0001) treatment than cells exhibiting high *FLT1* methylation. After combination treatment with DAC plus sunitinib (DAC + sunitinib: *t* = −3.40, *p* = 0.0011) or axitinib (DAC + axitinib: *t* = −5.87, *p* < 0.0001), cells exhibiting high *FLT1* methylation exhibited greater proliferation inhibition than cells with low *FLT1* methylation. In contrast, RCCs having low or high *KDR* methylation did not exhibit significant differences in proliferation inhibition after combination treatment with DAC and sunitinib (*t* = 1.89, *p* = 0.0620), DAC and axitinib (*t* = −1.23, *p* = 0.2240), or treatment with sunitinib (*t* = 0.99, *p* = 0.3242) or axitinib (*t* = −0.52, *p* = 0.6040) alone.

### Effects of *FLT1* or *KDR* knockdown on the sensitivity of RCCs to anti-VEGF/VEGFR drugs

**Fig. 6:**
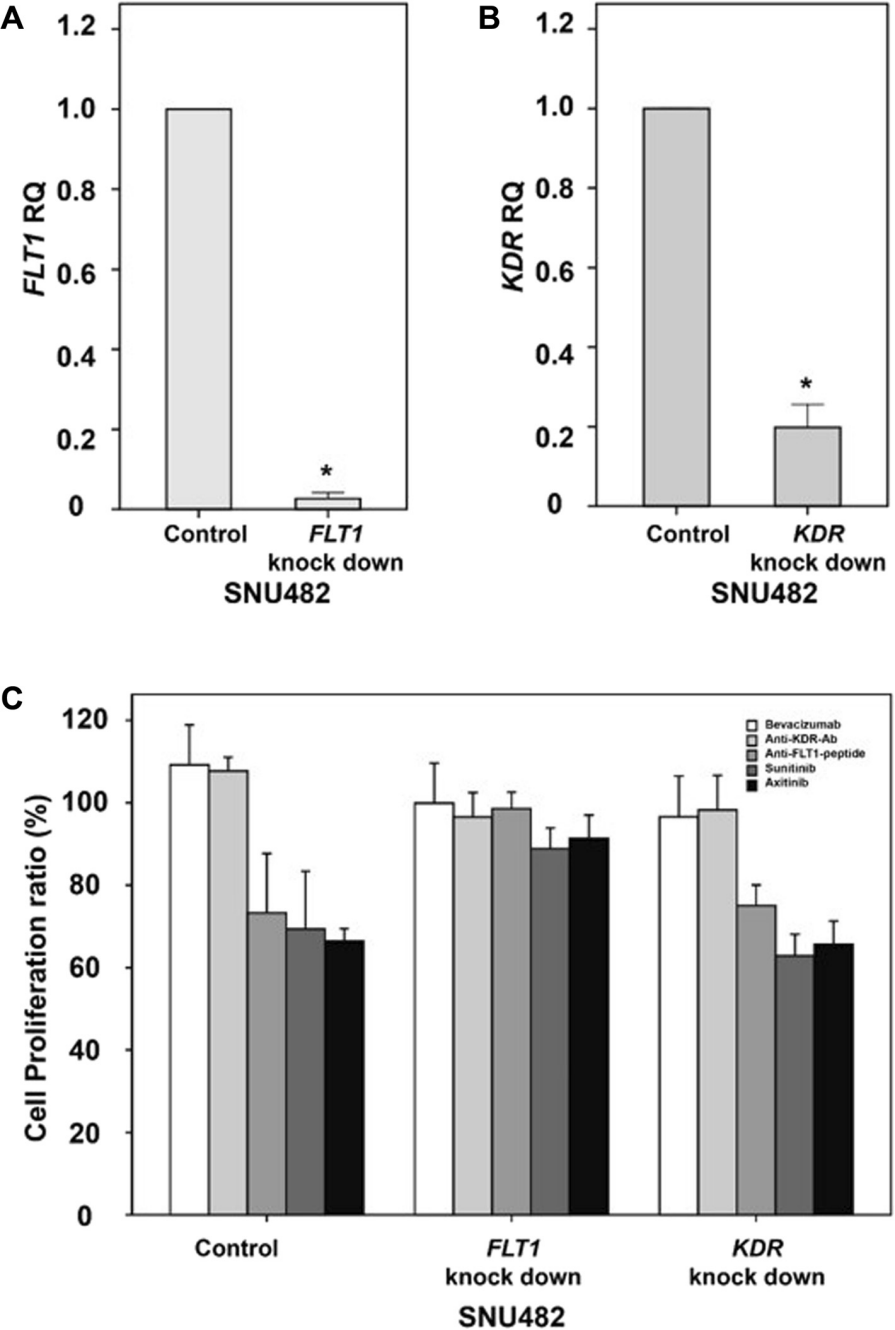
Expression changes and anti-VEGF/VEGFR drug efficacies following knockdown of *FLT1* or *KDR* in vitro. Expression of *FLT1* (**a**) and *KDR* (**b**) in SNU482 renal cancer cells after transduction with an shRNA targeting *FLT1* or *KDR*. The effects of bevacizumab, an anti-FLT1 peptide, an anti-KDR antibody, sunitinib, and axitinib on proliferation were evaluated in SNU482 cells transduced with lentiviral vectors expressing *FLT1* or *KDR* or an empty lentiviral vector (**c**). The *error bars* show the standard errors

*FLT1* expression (RQ) was significantly decreased to 0.027 (*t* = 131.53, *p* = 0.000) in *FLT1*-knockdown SNU482 cells transduced with a lentiviral vector expressing a short hairpin RNA (shRNA) against *FLT1* (Fig. 6a). Similarly, *KDR* expression (RQ) was significantly decreased to 0.198 (*t* = 45.75, *p* = 0.000) in *KDR* knockdown cells (Fig. 6b).

Analysis of proliferation revealed that the proliferation inhibition of control SNU482 cells transduced with an empty lentiviral vector was increased after treatment with the anti-FLT1 peptide, sunitinib, or axitinib (Fig. 6c). Similar proliferation inhibition was observed following treatment with the anti-FLT1 peptide, sunitinib, or axitinib in *KDR* knockdown cells, as observed with the control SNU482 cells. However, none of these drugs affected proliferation in *FLT1*-knockdown cells (Fig. 6c).

Two-factor analysis was used to evaluate the relationship between *FLT1* and *KDR* knockdown with the proliferation-inhibitory effects of the five anti-VEGF/VEGFR drugs. In *FLT1*-knockdown SNU482 cells, the growth inhibition by the anti-FLT1 peptide (*t* = 6.04, *p* < 0.0001), sunitinib (*t* = 6.87, *p* < 0.0001), and axitinib (*t* = 5.09, *p* < 0.0001) were significantly decreased, while that in cells treated with bevacizumab (*t* = −1.09, *p* = 0.2766) and anti-KDR antibody (*t* = −1.34, *p* = 0.1818) did not change. In *KDR* knockdown SNU482 cells, none of the drugs tested affected the cell proliferation (bevacizumab: *t* = −1.81, *p* = 0.0735; anti-KDR-antibody: *t* = −0.99, *p* = 0.3257; anti-FLT1 peptide: *t* = 0.28, *p* = 0.7794; sunitinib: *t* = −1.46, *p* = 0.1483; axitinib: *t* = −0.26, *p* = 0.7961).

### Methylation statuses of *VEGF*, *FLT1*, and *KDR* in patients with RCC treated with sunitinib

**Fig. 7:**
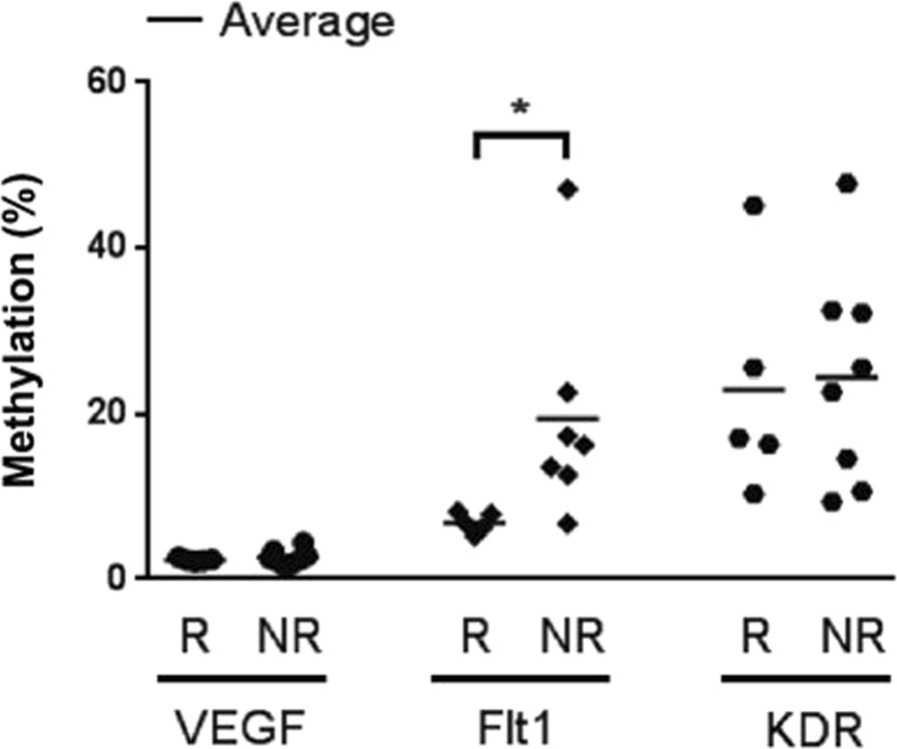
*VEGF*, *FLT1*, and *KDR* methylation in responder and nonresponder patients with renal cancer receiving sunitinib. An analysis of *VEGF*, *FLT1*, and *KDR* methylation statuses is shown for responders and nonresponders. *NR* nonresponder, *R* responder, ^*^ *p* < 0.05

To evaluate whether the methylation statuses of *VEGF*, *FLT1*, or *KDR* of renal cancer tissues are related to patient responses to sunitinib, we compared the methylation statuses of these targets in cancer tissues from 13 patients with RCC who were treated with sunitinib. The baseline characteristics of these patients are listed in Additional file 1: Table S1. Among these patients, five were responders (complete response, *n* = 1; partial response, *n* = 4), as assessed by the Response Evaluation Criteria in Solid Tumors (RECIST) criteria [6] and eight patients were nonresponders (stable disease, *n* = 5; progressive disease, *n* = 3). The responses to treatment were confirmed after 2 or 3 cycles of sunitinib, and the duration responses ranged from 9.7 to 22.8 months (Additional file 2: Table S2). The methylation statuses of *VEGF* (2.34 ± 0.21 % and 2.64 ± 0.91 % for responders vs. nonresponders, respectively; *p* = 0.240) and *KDR* (22.88 ± 13.59 % and 24.41 ± 13.05 % for responders vs. nonresponders, respectively; *p* = 0.422) were not significantly different between responders and nonresponders. However, *FLT1* promoter methylation was significantly higher in nonresponders (6.84 ± 1.19 % and 19.48 ± 13.15 % for responders vs. nonresponders, respectively; *p* = 0.030; Fig. 7).

## Discussion

In this study, we examined the effects of *FLT1*, *KDR*, and *VEGF* promoter methylations and expressions on the efficacy of anti-VEGF/VEGFR drugs in RCCs. The results provided pharmacoepigenomic evidence indicating that FLT1 is necessary to achieve effective inhibition of cell proliferation by drugs targeting the VEGF-VEGFR signaling axis in RCCs.

In this study, we found that anti-VEGF/VEGFR drugs inhibited cancer cell proliferation through mechanisms other than the inhibition of angiogenesis [2]. In previous studies, anti-VEGF/VEGFR drugs targeting extracellular VEGF (bevacizumab) [7], VEGFRs (the anti-FLT1 peptide [8] or anti-KDR antibody [9]), or intracellular VEGFR signaling (VEGF-specific TKIs) [10] were shown to have anti-angiogenic effects. In the present study, however, drugs targeting FLT1 (anti-FLT1 peptide) or intracellular VEGFR signaling (sunitinib and axitinib) showed significant proliferation inhibition in RCCs having no epigenetic alteration of *FLT1*. These findings suggested that the efficacies of various anti-VEGF/VEGFR drugs may differ based on the specific target within the VEGF-VEGFR signaling pathway in RCCs. In particular, VEGF-FLT1 and intracellular VEGFR signaling are important targets required for achieving successful inhibition by anti-VEGF/VEGFR drugs in RCCs. Furthermore, decreased proliferation inhibition was observed after *FLT1* knockdown in an RCC lacking methylation of both *FLT1* and *KDR*. These data indicated that the various anti-VEGF/VEGFR drugs tested inhibit cell proliferation through different targets within the VEGF-VEGFR signaling pathway in cancer cells. In particular, a lack of epigenetic alterations of the *FLT1* promoter may be the most important factor to consider when predicting the efficacy of anti-VEGF/VEGFR drugs in RCCs.

The epigenomic evidence provided in the present study showed that epigenetic gene silencing of *FLT1* caused by promoter hypermethylation was associated with insufficient inhibition of RCC proliferation by anti-VEGF/VEGFR drugs. Although epigenetic alterations have been reported as a mechanism contributing to cancer development [11], the therapeutic applications of epigenetic alterations in cancer treatment are not yet known [12]. Results from the present study showed that the epigenetic alteration of *FLT1*, but not *KDR*, was related to insufficient inhibition of proliferation by anti-VEGF/VEGFR drugs, particularly drugs targeting FLT1 and intracellular VEGFR signaling. Synergistic inhibition of RCC proliferation after combination treatment with a demethylating agent and sunitinib or axitinib (VEGF-TKIs) supported the importance of epigenomic *FLT1* modification in the efficacy of anti-VEGF/VEGFR drugs.

The present pharmacoepigenomic evidence suggested that *FLT1* could be a useful biomarker for predicting the efficacy of anti-VEGF/VEGFR drugs targeting FLT1 and intracellular VEGFR signaling. In the clinical setting, the anti-cancer effects of anti-VEGF/VEGFR drugs are unsatisfactory, despite promising results from several in vitro and in vivo experimental studies [2]. Researchers have suggested that these limited effects of anti-VEGF/VEGFR drugs may be due to the heterogeneous nature of tumor structures and the susceptibility of tumor blood vessels to anti-angiogenic therapy in different types of cancer [2]. However, VEGF is usually overexpressed in a variety of different types of cancer, including renal [13], breast [14], and ovarian cancers [15]. Moreover, paracrine-angiogenic and autocrine-mitogenic pathways function simultaneously to promote tumor growth [1]. While anti-VEGF/VEGFR drugs are thought to function via anti-angiogenic effects [3], antimitogenic effects may also be involved. The relationship between *FLT1* epigenetic alterations and anti-VEGF/VEGFR drug efficacy suggested that the *FLT1* methylation status could be used as a biomarker to predicting the success of these drugs and as an epigenetic mechanism to understand the failure of these drugs in some patients with renal cancer.

Additional studies are required to examine whether the present pharmacoepigenomic evidence can facilitate the development of appropriate clinical management strategies for patients with renal cancer. First, an evaluation of the lower limit of *FLT1* hypermethylation is needed to develop methods for predicting the efficacy of anti-VEGF/VEGFR drugs, particularly those targeting FLT1 or intracellular VEGFR signaling. In this study, we used a criterion of over 15 % methylation to define high methylation of both *FLT1* and *KDR* in RCC lines. In methylation analysis using tissues collected from the RCC patients of the present study, *FLT1* methylation showed a mean of 4 % in cancer tissues, even though that degree of methylation was significantly higher than observed in normal tissues from RCC patients. In contrast, renal cancer tissues from nonresponders to sunitinib had average of 20 % methylation at the *FLT1* promoter. Well-designed clinical and experimental studies are needed to verify the level of *FLT1* hypermethylation associated with lack of efficacy of anti-VEGF/VEGFR drugs. Additional clinical studies are also needed to evaluate the significance of *FLT1* hypermethylation in patients with cancer who have received anti-VEGF/VEGFR drugs as second-line drugs in combination chemotherapy. Previous studies have reported the efficacy of adding anti-VEGF/VEGFR drugs, such as bevacizumab [16] or VEGF-specific TKIs (PTK/ZK) [17], to other cancer drugs in the management of cancers. However, the additive effects of anti-VEGF/VEGFR drugs were variable. The role of *FLT1* hypermethylation in the efficacy of anti-VEGF/VEGFR drugs as supporting drugs has not been well characterized. Thus, evaluation of the function of *FLT1* hypermethylation in combination therapies should also be performed. Finally, the relevance of *FLT1* methylation for predicting the efficacy of anti-VEGF/VEGFR agents in other types of cancers should also be examined. *FLT1* methylation has been observed in lung, stomach, and colon cancer cells [4, 5]. In addition, the effects of VEGF-specific TKIs have been shown to vary according to the methylation status of *VEGFR* in other cancer types [4].

## Conclusions

The present study provided pharmacoepigenomic evidence to support the importance of *FLT1* in predicting the anti-cancer effects of anti-VEGF/VEGFR drugs. Intact VEGF-VEGFR signaling has been hypothesized to enable successful growth inhibition in cancer tissues [5]. Thus, our results may explain the importance of intact VEGF-VEGFR signaling in the efficacy of anti-VEGF/VEGFR drugs. In particular, VEGF-FLT1 signaling may be an important target for anti-VEGF/VEGFR therapy in patients with cancer. Therefore, the promoter methylation status of *FLT1* could be a useful biomarker to anticipate successful effects of anti-VEGF/VEGFR drugs targeting FLT1 or the intracellular tyrosine kinase activity of VEGFR.

## Methods

### Cancer cell lines and tissues

The SNU1 cell line was used as a control cell line and showed hypermethylation of both the *FLT1* and *KDR* promoters. H460 cells were used as another control cell line, which lacked methylation of both genes, and HUVECs were used as a control cell line to test the effects of anti-VEGF/VEGFR drugs on proliferation. Six RCC lines (A498, A704, ACHN, CAKI1, CAKI2, and SN12C) were maintained in Dulbecco’s Modified Eagle’s Medium (Gibco, Grand Island, NY, USA), while six other RCC lines (SNU1272, SNU228, SNU333, SNU349, SNU482, and SNU267) and the control cell lines (SNU1 and H460) were maintained in Roswell Park Memorial Institute (RPMI) 1640 medium (Gibco). The RCC line SN12PM6 was maintained in Modified Eagle Medium (Gibco). All media used for maintaining cancer cell lines contained 10 % fetal bovine serum (FBS, Gibco). HUVECs were grown in EGM-2 MV Endothelial Growth Medium (EGM-2 MV Bulletkit; Lonza Walkersville, Walkersville, MD, USA) for cell proliferation inhibition assays or otherwise maintained in EGM-2 Basal Medium (Lonza Walkersville) containing less than 2 % FBS. All RCC lines and SNU1 cells were purchased from the Korean Cell Line Bank (Seoul, South Korea), whereas H460 and HUVECs were purchased from the American Type Culture Collection (Manassas, VA, USA).

To evaluate epigenetic gene silencing in renal cancer tissues, we used normal and cancer tissues collected from eight renal cancer patients, which were provided by the Keimyung Human Bio-Resource Bank. The normal and cancer tissue samples were simultaneously collected within 30 min after their removal from each patient. Normal tissues were collected from the region separated over 2 cm from the cancer boundary. The tissues were stored in liquid nitrogen until DNA and RNA extractions were performed.

### Evaluation of the methylation statuses of *VEGF*, *FLT1*, and *KDR*

Promoter methylation of *VEGF*, *FLT1*, and *KDR* was evaluated by pyrosequencing analysis. Briefly, DNA and RNA from all tested cell lines and tissue samples (Table 1) were extracted using the AllPrep DNA/RNA Mini Kit (Cat. No. 80204; Qiagen, Valencia, CA, USA) following the manufacturer’s recommended protocol. DNA and RNA were also extracted from tissue samples using the AllPrep DNA/RNA Mini Kit, which involved homogenizing less than 20 mg of each sample in lysis buffer (Buffer RLT Plus, Cat. No. 1053393; Qiagen, Valencia, CA. USA) using the TissueLyser II (Qiagen, Valencia, CA, USA). Subsequently, lysates from each tissue sample were used for DNA and RNA extraction after a 3-min centrifugation step at maximum speed. Pyrosequencing of the *VEGF*, *FLT1*, and *KDR* genes was performed after bisulfite treatment of 1 μg of genomic DNA (Zymo EZ DNA Methylation Kit; Zymo Research, Irvine, CA, USA). PCR for pyrosequencing was performed in a total volume of 50 μL containing 1× buffer (67 mM Tris-HCl (pH 8.8), 6.6 mM MgCl_2_, 16.6 NH_4_SO_4_, and 10 mM 2-mercaptoethanol), 0.2 mM dNTP, 1 unit of Taq polymerase, 2 μL (100 ng) of bisulfite-treated DNA, and 0.1 mM of each forward and reverse primer, 1 of each primer pair being biotinylated (B): (*VEGF*: VEGF-pyro-F, (B)-5'-TAGGGAAGTTGGGTGAATGGA-3' and VEGF-pyro-R, 5′-TCCTAAAATAACCCCTAACCTTCT-3′; *FLT1*: FLT1-pyro-F, 5′-ATGGGTAGGAGGAGGGGTAA-3′ and FLT1-pyro-R, (B)-5'-TCCCCACCTACCCTCTTCTT-3', and *KDR*: KDR-pyro-F, 5′-GAGGGTGTAGGTAGGAGAGGATATTTAG-3′ and KDR-pyro-R, (B)-5′-CCCCCAAAAAACCATCAATATATAATC-3′). After a hot start, the PCR cycling conditions for each tested gene were as follows: 45 cycles of 95 °C for 30 s, annealing at varying temperatures (62 °C for *VEGF*, 63 °C for *FLT1*, and 64 °C for *KDR*) for 30 s, and 72 °C for 30 s. Pyrosequencing and methylation quantification of the biotinylated PCR product were performed using a sequencing primer for each gene (*VEGF*: VEGF-pyro-s, 5′-CCCCTAACCTTCTCCC-3′; *FLT1*: Flt1-pyro-s, 5′-GGATAAAGATTTTGAATT-3′; *KDR*: KDR-pyro-s, 5′-GGAGAGGATATTTAGGTTG-3′) and Pyro Gold CDT Reagents (Qiagen), using a PSQ HS 96 Pyrosequencing System (Qiagen) [5]. Primers for the pyrosequencing were designed based on the promoter sequences of *VEGF* (GenBank Accession Number M63971), *FLT1* (Accession Number D64016), and *KDR* (Accession Number X89776), shown in Fig. 1a [4]. We used mean values from all pyrosequenced CpG sites (3 adjacent CpG sites for *VEGF*, 10 for *FLT1*, and 4 for *KDR*) to determine the methylation levels for each gene (Fig. 1b) [4].

### Evaluation of *VEGF*, *FLT1*, and *KDR* expression

The expression levels of *VEGF*, *FLT1*, and *KDR* were evaluated using real-time RT-PCR [4]. Briefly, 2 μg of total RNA was transcribed using the High Capacity cDNA RT Kit (Applied Biosystems, Foster City, CA, USA). Real-time RT-PCR was performed in a Step-One Real-Time PCR System (Applied Biosystems) using a 20× TaqMan® Gene Expression Assay (Applied Biosystems) for *VEGF* (Hs00900054_m1), *FLT1* (Hs01052936_m1), *KDR* (Hs00176676_m1), or *β-actin* (Hs99999903_m1) as an endogenous control. The reaction mixtures for real-time RT-PCR contained 10 μL of TaqMan Universal Master Mix (Applied Biosystems), 1 μL of 20× primer probe mix, 7-μL distilled water, and 2-μL cDNA. Amplification was performed by denaturation for 10 min at 95 °C, followed by 40 cycles of 95 °C for 30 s, 60 °C for 30 s, and 72 °C for 30 s. Reactions were performed in triplicate for each gene. The expression difference of each target gene was evaluated using the ΔC_T_ method, normalizing C_T_ values a target genes to that of *β-actin*.

### Analysis of clear cell RCC data from TCGA

To evaluate relationships between the expression and methylation of *VEGF*, *FLT1*, and *KDR* using TCGA data, we used the cBioPortal for Cancer Genomics (www.cbioportal.org) online tools [18, 19]. First, Kidney Renal Clear Cell Carcinoma (TCGA, Provisional) data were selected among the listed cancer studies of the cBioPortal site. Then, we queried mutation frequencies and relationships between DNA methylation and gene expression of *VEGFA*, *FLT1*, and *KDR* in 320 samples selected from the Kidney Renal Clear Cell Carcinoma dataset.

### Proliferation assays after treatment with anti-VEGF/VEGFR drugs

The effects of an anti-VEGF antibody (bevacizumab, expressed in HEK293E cells and purified on a protein A-Sepharose column), an anti-KDR antibody (Cat. No. MAB3572, R&D Systems; Minneapolis, MS, USA), an anti-FLT1 peptide (Gly-Asn-Gln-Trp-Phe-Ile; synthesized by Peptron, Inc., Daejeon, South Korea) [8], and 2 VEGF-TKIs (sunitinib (Cat. No. PZ0012, Sigma-Aldrich, St. Louis, MO, USA) and axitinib (Cat. No. PZ0193, Sigma-Aldrich)) on cell proliferation were examined in vitro. HUVECs, SNU1 cells, H460 cells, and RCC lines were treated for 72 h with bevacizumab (2 μg/mL) an anti-FLT1 peptide (100 μM), an anti-KDR antibody (4 μg/mL), sunitinib (2 μM), or axitinib (2 μM). Untreated cells were used as controls. Proliferation was then examined using CCK8 assays (Dojindo Molecular Technologies, Inc., Rockville, MD, USA) following the manufacturer’s instructions. Briefly, 1 × 10^4^ cells were seeded in individual wells of 96-well plates (two wells per cell line) and incubated in growth media at 37 °C and 5 % CO_2_. After 24 h, the appropriate drug was added to one of the two wells, and the cells were grown for an additional 72 h. The optical density (OD) of each well was then measured to evaluate cell proliferation in each well. The proliferation assays were performed in three independent experiments. The average ODs of control or treated cells from three replicate assays were used to determine changes in proliferation after the 72-h treatment with anti-VEGF/VEGFR drugs. Changes in proliferation were determined using the ratio (%) of the average OD observed with treated cells to that observed with untreated cells.

### Evaluation of *VEGF*, *FLT1*, and *KDR* expression after demethylation

For demethylation experiments, four RCC lines (SNU482, having low methylation of both *FLT1* and *KDR*; SN12C, having high *FLT1*/low *KDR* methylation; ACHN, having low *FLT1*/high *KDR* methylation; and SNU333, having high methylation of both *FLT1* and *KDR*) and two control cell lines (SNU1 and H460) were used. The cell lines were treated with or without 5 μM DAC (Sigma) each day for 7 days [20]. Changes in the methylation of *VEGF*, *FLT1*, and *KDR* were compared between untreated and DAC-treated cells by pyrosequencing. Comparison of each target gene expression was first normalized to endogenous gene expression (ΔC_T_ = C_T(target gene)_ – C_T(β-actin)_) and then to the reference sample (ΔΔC_T_ = ΔC_T(DAC)_ – ΔC_T(No-DAC)_) in each tested cell line. Finally, the RQ of each gene was determined for each tested cell line using the formula, RQ = 2^−ΔΔC^_T_.

### Proliferation assay after demethylation and sunitinib treatment

To evaluate potential synergistic effects of sunitinib treatment after demethylation, control cells (SNU1 and H460) or RCC lines were treated with 5 μM DAC, 2 μM sunitinib, 2 μM axitinib, or a combination of DAC and sunitinib or axitinib. For the proliferation assays, 1 × 10^4^ cells were seeded in each of two wells of 96-well plates and incubated in growth medium at 37 °C and 5 % CO_2_. After 24 h, DAC, sunitinib, or axitinib was added to one well. For the combined treatment, DAC was added on the first day, and sunitinib or axitinib was added after 24 h. Cells were then incubated under these conditions for an additional 72 h. The OD of each well was measured to evaluate the proliferation status of the cells in each well. Proliferation assays were replicated three times for the control and treated cells. Changes in proliferation were determined using the ratio (%) of the average OD observed with treated cells to that observed with untreated cells at 72 h.

### Effects of *FLT1* or *KDR* knockdown on proliferation

SNU482 cells, having low methylation of both *FLT1* and *KDR* (Table 1), were used for in vitro knockdown assays following lentiviral vector-mediated transduction of shRNAs for *FLT1* (FLT1 MISSION shRNA Lentiviral Transduction Particles; TRCN0000194670; Sigma-Aldrich) or *KDR* (KDR MISSION shRNA Lentiviral Transduction Particles; TRCN0000199129; Sigma-Aldrich). On day 1, 1.6 × 10^4^ SNU482 cells were seeded in RPMI medium in separate wells of a 96-well plate. On day 2, the medium of each well was removed and replaced with 110 μL of medium containing 8 μg/mL hexadimethrine bromide. The plate was swirled gently to mix the well contents, and 2 μL of 10^6^ transducing units of lentiviral particles for each gene was added to the appropriate wells. The plate was then incubated for 20 h at 37 °C in a humidified incubator in an atmosphere of 5–7 % CO_2_. On day 3, the medium containing lentiviral particles was removed from the wells and replaced with 120 μL of fresh medium. On day 4, the medium was removed and replaced with fresh medium containing 2 μg/mL puromycin. The medium was replaced every 3–4 days thereafter. Proliferation inhibition assays were performed after treatment with bevacizumab, an anti-KDR-antibody, an anti-FLT1-peptide, sunitinib, and axitinib, as described above.

### Analysis of the effects of methylation statuses of *VEGF*, *FLT1*, and *KDR* on sunitinib sensitivity in patients with renal cancer

We reviewed data sets from 21 patients who were treated with sunitinib for metastatic renal cancer between September 2007 and December 2009 [21]. The inclusion criteria were as follows: histologically confirmed clear cell RCC, measurable lesions by computed tomography or magnetic resonance imaging, and a performance status of 0–2 as assessed by the Eastern Cooperative Oncology Group criteria [22] and the number of risk factors as defined by the Memorial Sloan-Kettering Cancer Center Risk Group [23]. Patients with brain metastases were excluded. The objective clinical response (complete response, partial response, stable disease, or progressive disease) was assessed using the RECIST criteria [6]. For analysis of the methylation status of *VEGF*, *FLT1*, and *KDR*, tumor tissues from 13 patients were studied. The study protocol (File No.: 2014-04-030) was approved by the Institutional Review Board of Chungnam National University Hospital. The requirement for informed consent from the patients was waived because of the retrospective nature of this study.

To evaluate the methylation status in renal cancer tissues, DNA was extracted from paraffin-embedded cancer tissues from each patient using the EpiTect Plus FFPE Lysis Kit (Qiagen). After bisulfite treatment of the DNA (EpiTect Plus DNA Bisulfite Kit; Qiagen), the promoter methylation status of *VEGF*, *FLT1*, and *KDR* for each patient was evaluated by pyrosequencing. The methylation statuses of these three genes were compared between responders (complete response or partial response) and nonresponders (stable disease or progressive disease).

### Statistical analysis

Differences in gene expression (ΔC_T_) according to changes in promoter methylation were analyzed using correlation analysis. Changes in gene expression (RQ) after the in vitro knockdown of *FLT1* or *KDR* were compared using paired *t* tests. A GLMM was used to analyze whether *FLT1* or *KDR* methylation status was associated with inhibited proliferation following treatment with anti-VEGF/VEGFR drugs in all tested RCC lines with different *FLT1* or *KDR* methylation statuses. For the GLMM, we assumed that each RCC line was randomly selected from each RCC line group classified according to *FLT1* or *KDR* promoter methylation status. Three-factor analysis was performed to evaluate whether the demethylation of *FLT1* or *KDR* synergistically enhanced the effects of sunitinib or axitinib treatment in four RCC lines with different *FLT1* and/or *KDR* methylation statuses. Two-factor analyses relating the knockdown status of *FLT1* or *KDR* and the inhibitory effects of the five tested drugs were performed to evaluate potential statistical interactions. Differences in methylation in *VEGF*, *FLT1*, and *KDR* between normal and cancer tissues collected from renal cancer patients were compared using Wilcoxon’s singed-rank test. The relationship between gene expression and methylation changes in the renal cancer tissues was also analyzed using correlation analysis between the reciprocal of the percent methylation and the RQ values observed in cancer tissues. To compare differences in *FLT1* and *KDR* promoter methylation levels between responders and nonresponders in different groups of renal cancer patients, we used a paired *t* test. All statistical analyses were performed using SPSS software, version 20.0 (IBM, Chicago, IL, USA) and SAS software, version 9.3 (SAS, Cary, NC, USA).

## Additional files

Additional file 1: Table S1. Baseline characteristics of 13 advanced RCC patients treated with sunitinib. (DOCX 14.9 kb) Additional file 2: Table S2. Characteristics of five advanced RCC patients who responded to sunitinib treatment. (DOCX 14.2 kb)

## Abbreviations

DAC: 5-aza-2′-deoxycytidine
HUVEC: human umbilical vein endothelial cell
OD: optical density
RCC: renal cancer cell
RECIST: Response Evaluation Criteria in Solid Tumors
RPMI: Roswell Park Memorial Institute medium
RQ: relative quantity
RT-PCR: reverse transcription polymerase chain reaction
VEGF: vascular endothelial growth factor
VEGFR: vascular endothelial growth factor receptor
