# Supplementary material for: Decreased efficacy of drugs targeting the vascular endothelial growth factor pathway by the epigenetic silencing of FLT1 in renal cancer cells
Source: Clin Epigenetics. 2015 Sep 16;7:99. doi: 10.1186/s13148-015-0134-9 (PMC4572656; doi:10.1186/s13148-015-0134-9)
Supplement: Additional file 1: Table S1. — Baseline characteristics of 13 advanced RCC patients treated with sunitinib. (DOCX 14.9 kb) [file 13148_2015_134_MOESM1_ESM.docx]

| Table S1. Baseline characteristics of 13 advanced RCC patients treated with sunitinib | | |
| --- | --- | --- |
| Characteristic | No. | % |
| Age, years  Median  Range | 67  57-75 | |
| Sex  Male  Female | 12  1 | 92  8 |
| ECOG performance status  0  1  2 | 8  4  1 | 62  31  8 |
| Prior therapy  Nephrectomy  Cytokine | 11  5 | 85  38 |
| Lesions at baseline  Bone  Liver  Lung  Lymph node  Any other site | 4  2  9  4  4 | 31  15  69  31  31 |
| MSKCC risk group (No. of factor)  Favorable (0)  Intermediate (1,2)  Poor (≥3) | 3  8  2 | 23  62  15 |
| RCC, renal cell carcinoma; ECOG, Eastern Cooperative Oncology Group; MSKCC risk group, Memorial Sloan-Kettering Cancer Center risk group [23]; %, rounded. | | |
