# Supplementary material for: Decreased efficacy of drugs targeting the vascular endothelial growth factor pathway by the epigenetic silencing of FLT1 in renal cancer cells
Source: Clin Epigenetics. 2015 Sep 16;7:99. doi: 10.1186/s13148-015-0134-9 (PMC4572656; doi:10.1186/s13148-015-0134-9)
Supplement: Additional file 2: Table S2. — Characteristics of five advanced RCC patients who responded to sunitinib treatment. (DOCX 14.2 kb) [file 13148_2015_134_MOESM2_ESM.docx]

| Table S2. Characteristics of five advanced RCC patients who responded to sunitinib treatment | | | |
| --- | --- | --- | --- |
| Best Response | Duration of Response  (months)^†^ | No. of Cycle of Sunitinib Treatment  When Response Confirmed |  |
| CR  PR  PR  PR  PR | 22.8  9.7  15.9  21.7  21.6 | 2  3  2  3  3 |  |
| CR, complete response; PR, partial response. Individual patient’s responses were assessed by response evaluation criteria in solid tumor (RECIST). Time from first response to time of documented progression, death, or time to last tumor assessment. | | | |
